# Kv1.3 Channel, a Targetable Piece in the Complex Jigsaw Puzzle of Vascular Calcification?

**DOI:** 10.1093/function/zqaa049

**Published:** 2020-12-28

**Authors:** Maria F Gomez

**Affiliations:** Department of Clinical Sciences in Malmö, Lund University Diabetes Centre, Lund University, Sweden

## A Perspective on “Kv1.3 Channel Inhibition Limits Uremia-Induced Calcification in Mouse and Human Vascular Smooth Muscle”

In parallel with an unprecedented progress in vascular and stem cell biology during the past two decades, our understanding of vascular calcification has evolved from being perceived as a harmless, passive deposition of minerals to what we now consider a highly regulated, cell-mediated process that is strongly associated with chronic kidney disease (CKD) and confers increased risk for incident cardiovascular disease. A PubMed search of the term “vascular calcification” reveals an impressive exponential growth in the number of original articles published between 2000 and 2015, but this trend seems to have slowed down in the past 5–6 years. Studies have helped us understand that vascular calcification can engage a large number of key mediators and signaling pathways and that its anatomical and histological location can determine the type and severity of the clinical outcomes. Despite this significantly increased knowledge regarding the underlying pathophysiology, very little progress has been made when it comes to therapies and only a handful of interventional studies have documented any impact on vascular calcification other than modest effects in slowing down the process. The reasons for this lack of translational progress are many, including a somewhat conventional view of vascular calcification with most interventions aiming at restoring or reverting the uremic environment or targeting calcification too late in the process.

In this issue of *Function*, Cazaña-Pérez et al. provide new mechanistic insight in the regulation of vascular calcification,^1^ directing our attention to the vascular smooth muscle cell (VSMC), one of the main drivers in both medial and intimal calcification.^2^ The authors ask whether the transdifferentiation of VSMCs into an osteoblast-like or calcific secretory phenotype that takes place early in the process and is central in the pathophysiology of vascular calciﬁcation,^3^ is associated with changes in ion-channel expression. Indeed, upon interrogation of 92 genes coding for relevant ion channel subunits (ie, K^+^ channels, voltage-dependent Ca^2+^ channels, epithelial Na^+^ channels, Cl^−^ channels, and TRP channels), extensive changes in the pattern of ion channel expression were observed when VSMCs were exposed to a uremic milieu. One of the most dramatic changes observed was the induction of the voltage-dependent K^+^ channel Kv1.3, a channel that had been previously associated with proliferation and phenotypic modulation of VSMC.^^4–6^^ This induction of Kv1.3 channels functionally translated into increased contribution of this channel to outward K^+^ currents. The authors then go on to show, using both murine and human intact vascular preparations, that inhibition of Kv1.3 efficiently limits uremia-induced phosphate deposition in the arterial wall. Considering the pharmacological advantages of targeting ion channels due to their relatively easy accessibility, these findings are certainly worth following up, especially given the need for new therapeutic venues for treating vascular calcification.

Several aspects of this study are worth highlighting, such as the use of a recently established in vitro calcification and osteochondrogenic transdifferentiation model based on the exposure of human aortic smooth muscle cells to serum from uremic patients.^7^ This model is characterized by the rapid calcification, upregulation of osteochondrogenic markers, and phenotypic remodeling of viable and proliferative VSMCs, recapitulating many of the changes so far described to occur in vivo (reviewed in Nelson et al.^8^). The use of this cellular model in combination with experiments using intact human and murine arteries, and both pharmacological and genetic manipulation of Kv1.3 are merits of this study.

Another important observation from this study is that the pattern of ion channel expression triggered by the uremic milieu in a VSMC calcific phenotype seems to be distinct from that described by the same group as being the fingerprint for a VSMC proliferative phenotype. Can this calcific ion channel expression pattern be used as an early biomarker predictive of vascular calcification?

**Figure 1.**
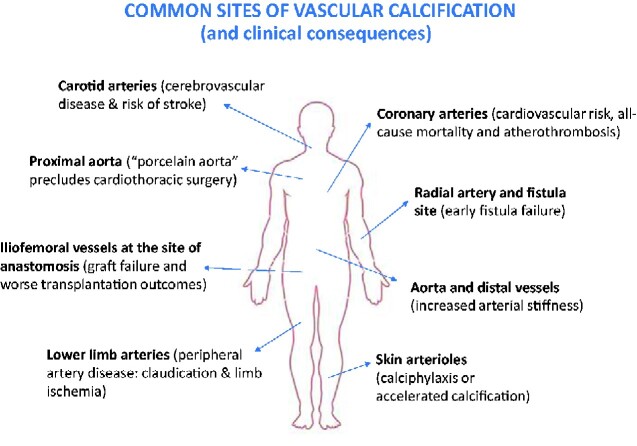
Variable Anatomical Distribution and Clinical Presentation of Vascular Calcification.

CKD remains a major global public health problem and has significant impact on patient mortality, morbidity, and quality of life. CKD is estimated to affect 10%–15% of the population and contribute to 5–10 million deaths annually, having significant consequences on health care costs.^9^^,^^10^ Even though treatments have improved for diabetes and hypertension, the first and second leading causes of CKD, the total CKD disease burden is still increasing. CKD patients exhibit a 2- to 5-fold higher rate of vascular calcification than that of age-matched non-CKD patients. Figure 1 shows the most common sites of vascular calcification, illustrating a very variable anatomical distribution and clinical presentation. Histologically, vascular calcification can affect different layers of the arterial wall (intima, media, or both). This distinct localization of vascular calcification sites could be exploited therapeutically. It would be interesting to test whether local delivery or topic application of Kv1.3 inhibitors, for example, to the fistula site could prevent early fistula failure.

It is clear that vascular calcification is not a single entity, but instead the result of a wide range of biological processes, including but not limited to those related to (1) phosphate, bone and mineral metabolism, (2) decreased calciﬁcation inhibitors (ie, Matrix Gla protein, Fetuin-A, Klotho [or FGF-23], Pyrophosphate, Osteopontin), (3) increased calcification promoters (ie, Osteocalcin, Alkaline phosphatase, Inﬂammatory cytokines, Runx2), (4) phenotypic transdifferentiation of VSMCs which is the focus of Cazaña-Pérez et al. (5) procalciﬁc matrix vesicles, (6) uremic milieu, (7) cellular apoptosis, (8) procalciﬁc miRNAs.^8^ Vascular calcification can also be the consequence of pharmacological interventions, with examples of therapies that are associated with an increase in vascular calcification but that have opposite effects on cardiovascular risk (ie, calcium-containing phosphate binders and statins). This last example highlights that there is no straightforward linear association between the amount of vascular calcification and risk for clinical events.^10^ Cazaña-Pérez et al. introduce KV1.3 channels as regulators of vascular calcification. Additional studies will be needed to understand the mechanisms linking Kv1.3 to cell calcification and whether Kv1.3 signaling interacts with other previously identified biological processes.

## Funding

M.F.G. receives funding from the Swedish Heart and Lung Foundation [#20160872]; the Swedish Research Council [#2018-02837; #2014-03352; EXODIAB #2009-1039], and the Swedish Foundation for Strategic Research (LUDC-IRC #15-0067). Also, from the Innovative Medicines Initiative 2 Joint Undertaking under grant agreement no. 115974 (BEAt-DKD). This Joint Undertaking receives support from the European Union’s Horizon 2020 Research and Innovation Programme and European Federation of Pharmaceutical Industries and Associations with JDRF.

## Conflicts of Interest Statement

None declared.
